# Potential of Olfactory Ensheathing Cells from Different Sources for Spinal Cord Repair

**DOI:** 10.1371/journal.pone.0062860

**Published:** 2013-04-24

**Authors:** Anne Mayeur, Célia Duclos, Axel Honoré, Maxime Gauberti, Laurent Drouot, Jean-Claude do Rego, Nicolas Bon-Mardion, Laetitia Jean, Eric Vérin, Evelyne Emery, Sighild Lemarchant, Denis Vivien, Olivier Boyer, Jean-Paul Marie, Nicolas Guérout

**Affiliations:** 1 UPRES EA 3830, Institute for Research and Innovation in Biomedicine, University of Rouen, Rouen, Normandy, France; 2 Otorhinolaryngology, Head and Neck Surgery Department, Rouen University Hospital, Rouen, Normandy, France; 3 Inserm UMR-S 919, Serine Proteases and Pathophysiology of the Neurovascular Unit, GIP Cyceron, Université de Caen Basse-Normandie, Caen, France; 4 Inserm, U905, Institute for Biomedical Research and Innovation, University of Rouen, Rouen, Normandy, France; 5 Platform of Behavioural Analysis (SCAC), Institute for Research and Innovation in Biomedicine, Rouen University, France, National Center of Scientific Research (CNRS) - DR19, France; 6 Rouen University Hospital, Department of Immunology, Rouen, Normandy, France; University of Milan-Bicocca, Italy

## Abstract

Spinal cord injury (SCI) induces a permanent disability in patients. To this day no curative treatment can be proposed to restore lost functions. Therefore, extensive experimental studies have been conducted to induce recovery after SCI. One of the most promising therapies is based on the use of olfactory ensheathing cells (OECs). OECs can be obtained from either the olfactory bulbs (OB-OECs) or from olfactory mucosa (OM-OECs), involving a less invasive approach for autotransplantation. However the vast majority of experimental transplantations have been focusing on OB-OECs although the OM represents a more accessible source of OECs. Importantly, the ability of OM-OECs in comparison to OB-OECs to induce spinal cord recovery in the same lesion paradigm has never been described. We here present data using a multiparametric approach, based on electrophysiological, behavioral, histological and magnetic resonance imaging experiments on the repair potential of OB-OECs and OM-OECs from either primary or purified cultures after a severe model of SCI. Our data demonstrate that transplantation of OECs obtained from OB or OM induces electrophysiological and functional recovery, reduces astrocyte reactivity and glial scar formation and improves axonal regrowth. We also show that the purification step is essential for OM-OECs while not required for OB-OECs. Altogether, our study strongly indicates that transplantation of OECs from OM represents the best benefit/risk ratio according to the safety of access of OM and the results induced by transplantations of OM-OECs. Indeed, purified OM-OECs in addition to induce recovery can integrate and survive up to 60 days into the spinal cord. Therefore, our results provide strong support for these cells as a viable therapy for SCI.

## Introduction

Spinal cord injury (SCI) is a devastating disease leading to a loss of neural conduction. Sensory and motor pathways are interrupted resulting to paraplegia or tetraplegia for patients. After SCI several mechanisms take place such as astrocyte reactivity, formation of the glial scar, invasion of inflammatory cells resulting in a lack of axonal regrowth [1], [2]. To this day no curative treatment is available for clinical use. Therefore extensive experimental studies have been conducted to increase axonal regrowth and to promote functional recovery after SCI using various therapies [3], [4]. Many of these approaches have used cellular transplantation, such as Schwann cells (SC), neural stem cells, induced pluripotent stem cells or olfactory ensheathing cells (OECs, “OECs” is used to name all the olfactory ensheathing cells) with contrasting results [5]–[12]. OECs have gained considerable interest for the past decade due to the fact that OECs have shown a great potential to restore both functional and anatomical recoveries after SCI [13]. OECs are neural crest cells which participate to neurogenesis occurring in the primary olfactory system throughout life in mammals [14]. Following transplantation in lesioned spinal cord, OECs were shown to enhance axonal regrowth, regulate astrocyte reactivity, increase angiogenesis and modulate glial scar leading to functional recovery in various models of SCI in rodents [15]–[19]. However, OECs are constituted of several subpopulations and the role of each of them following transplantation in injured spinal cord is not well described [20], [21]. Specifically, OECs can be obtained from two different regions: olfactory mucosa (OM) and olfactory bulbs (OB) and it appears that OECs obtained from OM (OM-OECs) and OB (OB-OECs) have not the same properties and effects after transplantation *in vivo* [22], [23]. Moreover, culture conditions, in particular the presence of microenvironment, can greatly influence OECs behaviors *in vitro* and *in vivo* [21], [24]. OM-OECs are closely associated with primary olfactory neurons and fibroblasts, whereas OB -OECs are in contact with fibroblasts and astrocytes both *in vitro* and *in vivo* [25]–[28]. For a clinical purpose these two aspects have some relevant consequences. Indeed, for clinical use, OECs could be obtained for autotransplantation easily by a simple biopsy of OM or more invasively, as described by Rubio et al., after an unilateral bulbectomy from OB [29]. Furthermore, OECs can be transplanted either as primary cultures or after additional purification steps and it is well described that time in culture can alter neurogenic properties of OECs [30], [31].

Here we have directly compared the potential of OECs obtained and purified from the two sources in a severe model of SCI by a multiparametric approach. We demonstrate that transplantation of OECs induces electrophysiological and functional recovery, reduces astrocyte reactivity and glial scar formation and improves axonal regrowth. Moreover, in our model, transplantation of OECs from OM represents the best benefit/risk source, providing support for these cells as a new therapy for SCI in human.

## Materials and Methods

### General Overview

**Figure 1:**
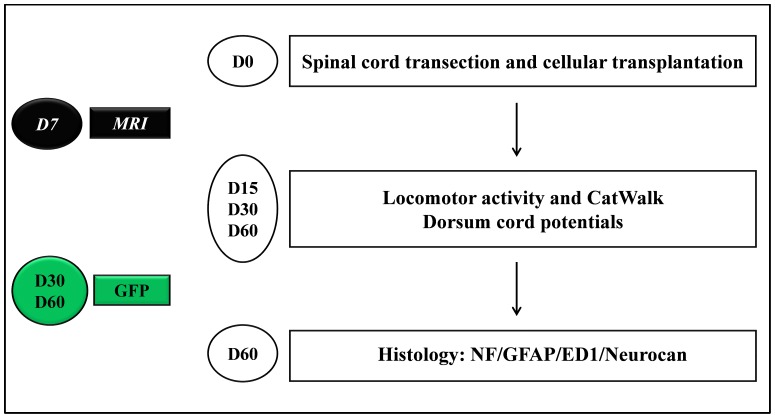
Experimental paradigm illustrating the timeline of the major experimental manipulations. Cells or medium were transplanted into sectioned spinal cords (**D0**). 15, 30 and 60 days after surgery animals were analyzed for electrophysiological and locomotor activities (**D15**, **D30**, **D60**). Histological analyses were performed 60 days after transplantation for all the groups (**D60**) (n = 10). On additional animals of OM-purified OECs tracking of GFP+ cells were performed 30 and 60 after transplantation (**D30**, **D60**). Each time point (n = 3) (in green).

An overview of the paradigms used in this study is presented in Figure 1.

Briefly, cellular transplantations were performed after complete transection of the spinal cord from two sources (OB and OM) and cultured in two different ways (primary and purified). For OB (Primary OB) and OM (Primary OM), primary cultures were transplanted after 5 days *in vitro* (DIV). For OB (Purified OB), purified cultures were transplanted after 10 DIV. For OM (Purified OM), purified cultures were transplanted after 14 DIV. All different cell types were transplanted immediately after spinal cord transection. All groups of animals were analyzed 15, 30 and 60 days after surgery for electrophysiological and locomotor activities. Histological analyses were performed blindly 60 days after transplantation for all the groups. Then, additional animals treated with Md, Primary OB and Purified OM, which showed higher potential to promote recovery, were used for MRI analyses 7 days after transplantation. Finally, OM purified cells, which constituted the most relevant group for clinical use, were labeled with GFP to visualize them 30 and 60 days after transplantation.

### Animals

Experiments were performed after acceptance by the local Ethics Committee for Animal Experiments in Normandy: N/02-11-10/22/11-13 in accordance with the French law on the protection of animals.

For this study, 95 8-weeks old and 30 3–4 weeks old male inbred Fischer rats were used (Charles River, L’Arbresle, France).

Individual rats were identified by implantation of glass tag (Biolog-id, Bernay, France); glass tag numbers have been used to perform blindly analyses.

### Cell Culture

All cultures have been performed in semi-sterile conditions with autoclaved surgery tools.

#### Primary cultures

OB and OM primary cultures were prepared as described previously with slight modifications [25], [26]. Briefly, rats were deeply anesthetized (isofluorane) and decapitated. OB from one rat were immediately dissected and placed in 1 ml of Hank’s buffered salt solution (H.B.S.S, Invitrogen, Carlsbad, CA), after removing the meninges. H.B.S.S containing 0.1% trypsin (Invitrogen) and OB were incubated for 45 min at 37°C. Trypsinization was stopped by adding Dulbecco’s Modified Eagle’s/Ham’s F12 medium (D.M.E.M/F12, Invitrogen), supplemented with 10% Fetal Bovine Serum (F.B.S, Invitrogen) and 1% penicillin/streptomycin (Invitrogen) (DF-10S). The tissue was centrifugated at 1.000 RPM for 3 min, resuspended in DF-10S and centrifugated again. Tissue was then triturated in 3 ml DF-10S using a micropipette, until a homogenous cell suspension was obtained. Prior plating, undigested tissue blocks were removed by pipetting. Then, cells were plated (1.5–3.10^6^ cells per flask) in DF-10S in 25 cm^2^ flasks (Starlab, Bagneux, France), pre-coated with poly-L-Lysine (50 µg/ml, Invitrogen). The flasks were incubated at 37°C, 5% CO_2_. The medium was changed every two days. Five days after plating, OB primary cultures were used for transplantation.

In the same rats, the nasal cavity was opened sagittally with a spatula and OM was cut in small pieces. Care was taken to avoid to take cartilage. After a short rinse in H.B.S.S, samples from 1 rat were digested enzymatically in 1 ml of collagenase A (0.5%) for 45 min at 37°C (Roche Diagnostics, Penzbeg, Germany).The OM is easily identified in the rat by its posterior position on the nasal septum and by the yellowish appearance of the epithelial surface. Care was taken to avoid the anterior edge of the OM which could have been contaminated with respiratory epithelium. Respiratory mucosa was removed from the dorso-anterior region of the septum. Then, samples were cultivated with the same protocol that for OB cultures [25]. Five days after plating, OM primary cultures were used for transplantation.

#### Olfactory ensheathing cells purification

OECs purification from OB were performed in using method described by Nash based on the differing rates of attachment of the various harvested cell types [32]. Briefly, the method of differential adhesion is based on a very low adhesion rate of OEC to the surface of uncoated culture flasks [32], [33]. The cell suspension obtained from OB was resuspended in DF-10S, seeded on uncoated flask and cultured at 37°C with 5% CO_2_. After 18 h, the supernatant was removed and re-plated on other uncoated flasks and incubated for additional 36 h. The supernatants from these flasks were removed and re-plated on 25 cm^2^ poly-L-lysine-coated culture flasks with DF-10S. Ten days after plating, OB purified cultures were used for transplantation.

OECs purification from OM was performed by using the method described by Bianco and modified by Gueye et al. [34], [35]. Briefly, after one week in primary culture, cells were split by trypsinisation and replated in serum-free culture medium supplemented with 25 ng/mL of transforming growth factor α (Sigma Aldrich, St Louis, MO) onto pre-coated flasks (poly-L-Lysine 50 µg/ml). The second passage was performed 1 week after. Fourteen days after plating, OM purified cultures were used for transplantation.

#### Preparation of the cells for grafting

Before transplantation, cultures were trypsinized to remove them from the dishes, and the cells were counted using a hemocytometer. Cultures cells were resuspended in DF-10S immediately before grafting for a final concentration of 100,000 cells/µl. Samples of the different cultures were prepared for characterization as described below.

#### Flow-cytometry analysis of OECs

In order to characterize cellular preparations, flow-cytometry analysis was conducted. The cells were washed with PBS and treated with trypsin/EDTA in conjunction with the use of a cell scraper to remove adherent cells. Then, cells were washed with PBS/EDTA and immunolabeled, during 15 min at 4°C, with the the anti-p75LNGFr (1∶100) (Chemicon, Billerica, MA.). At the end of the incubation, 2 ml of PBS/EDTA was added and the cells were centrifuged (300 g for 5 min). Cells were incubated with the secondary antibodies conjugated with phycoerythrin (1∶200) for p75 (BD Biosciences, San Jose, CA). At the end of the incubation, 2 ml of PBS/EDTA was added and the cells were centrifuged (300 g for 5 min). Then, cells were resuspended in 500 µl of PBS/EDTA. The total number of 10,000 events was analyzed on a FACSCalibur flow-cytometer (BD Biosciences) directly after the staining procedure. Data were processed using the FACSdiva program (BD Biosciences). Proportion of p75 positive cells in the various samples was determined by measuring the fluorescence intensity dot plot compared to the total amount of non-labeled cells.

**Figure 2:**
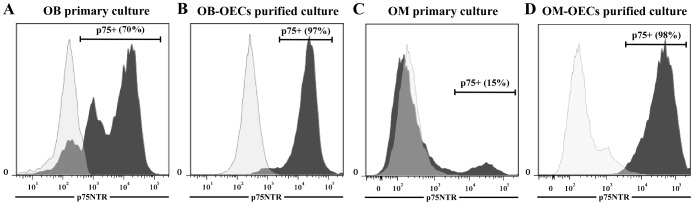
P75 expression of primary OB (A) and OM (C) and purified OB-OECs (B) and OM-OECs (D) cultures. After 5 and 10 days *in vitro*, cell surface expression of p75 in primary OB and purified OB cultures was determined by flow cytometry. Primary OB cultures were positive for p75 at 70% (**A**), whereas, OB purified cultures were positive for p75 at 97% (**B**). At the same time, After 5 and 14 days *in vitro* cell surface expression of p75, respectively, in primary OM and purified OM cultures was determined by flow cytometry. Primary OM cultures were positive for p75 at 15% (**C**), whereas, OM purified cultures were positive for p75 at 98% (**D**). Numbers indicate the total population and the percentage of cells present in the indicated gated region.

Flow cytometry analysis allowed to determinate that OB primary cultures contained approximately 70% of p75 positive cells (Figure 2A) and OB purified cultures contained approximately 97% of p75 positive cells (Figure 2B), whereas in the same time OM cultures contained about 15% of p75 positive cells (Figure 2C) and OM purified cultures contained approximately 98% of p75 positive cells (Figure 2D).

#### GFP labeling of transplanted cells

This evaluation was performed in 6 other rats for Purified OM group, 3 rats were used at each time point (30 and 60 days).

Briefly, five days before transplantation, OM-OECs purified cultures were infected with a lentiviral vector harboring enhanced GFP (multiplicity of infection: 20, time of exposure overnight). Efficiently of infection was 80% determined by flow cytometry as previously described in “materials and methods”. After several washing steps, GFP positive cells were injected as described below.

### Experimental Spinal Cord Lesion and Cell Transplantation Procedure

For experimental groups, adult Fischer inbred rats (225–250 g) were used.

All the rats were anesthetized by intraperitoneal injection of ketamine hydrochloride (12.5 mg/kg) and chlorpromazine hydrochloride (0.625 mg/kg).

Five groups (n = 10) were classified as follows:

Group 1: Medium group (Md). Complete spinal cord transection was performed and DF-10S was injected.

Group 2: OB-treated group (Primary OB). Complete spinal cord transection was performed and primary olfactory bulb cultures were transplanted.

Group 3: OM-treated group (Primary OM). Complete spinal cord transection was performed and primary olfactory mucosa cultures were transplanted.

Group 4: OB OECs-treated group (Purified OB). Complete spinal cord transection was performed and purified olfactory ensheathing cells from olfactory bulb were transplanted.

Group 5: OM OECs-treated group (Purified OM). Complete spinal cord transection was performed and purified olfactory ensheathing cells from olfactory mucosa were transplanted.

Control animals (Ctrl) were also used for histological analyses, for these no surgery was performed.

For all operated groups surgery was performed by the same operator as follow:

Through a midline dorsal incision, a standard bilateral Th10 laminectomy was performed with great care, in order to visualize the spinal cord. The dura was opened longitudinally and laterally at the level of the Th10 dorsal root. Then, the spinal cord was completely sectioned with a microscalpel, the cord stumps were lifted and a needle was passed between the two stumps to ensure completeness of the lesion.

Immediately after spinal cord transection, cells or medium were transplanted using a 5 µl Hamilton glass syringe, connected to a 27 gauge needle and attached to a micromanipulator. Md treated animals received injection of DF-10S, and transplanted animals received cellular preparations. Medium or cells were injected to the spinal cord on the site of the lesion. Injections were delivered at spinal sites 1.5 mm laterally from the midline (2 sites) and 2 mm depth: 1 mm rostrally and 1 mm caudally. Each animal received a total of 4 injections (4×1 µl) which contained cellular suspension (100,000 cells/µl) or DF-10S. The injection was given at 0.5 µl/min. The delivery needle was left in the injection site for 2 min to avoid reflux. No immunosuppressant was used.

All the rats were caged individually under heat lamp during 24 hours after surgery and give antibiotics for the first two weeks (Ceftriaxone, 80 mg/Kg).

Before and after the experiments, rats were kept on standard laboratory food and tap water ad libitum, with an artificial 12 hours light/dark cycle and caged by threes.

During all the experiments the bladder was compressed every day by manual abdominal pressure and rats were washed daily.

Despite all the precautions taken a high morbidity has been recorded, around 30%, thereby to obtain 10 rats in every group 15 rats per group were operated. 95 rats have been used for this study.

### Evaluation

Evaluations were performed 15, 30 and 60 days after surgery for electrophysiological and behavioral analyses and at 60 days after surgery for histological analysis on 10 rats per group.

Tracking of GFP positive OECs was performed at 30 and 60 days after surgery on 6 other rats for Purified OM treated group.

Experimental schedule is described in Figure 1.

#### Electrophysiological studies

Measurement of cord dorsum potentials (CDP) was recorded. For this, rats were anaesthetized with ketamine hydrochloride (30 mg/kg) in order to induce slight sedation. Then, an electrode was placed sub-cutaneaously in the abdomen of the rats for electrical isolation and CDP were recorded using two recording electrodes placed in gastrocnemius muscles separated by 5 mm. Firstly, spinal cord has been stimulated in sub-lesional to validate the record. Stimulations were performed at Th2 level (above the lesion site) using a magnetic stimulator (Magstrim, Rapid 2®) by 5 stimulations separated by 30 seconds with an intensity of 90% for supra maximal response. Then, amplitude was recorded and averaged for each animal.

#### Measurement of locomotor activity

Measurement of locomotor activity was performed as described previously [36]. Animals were placed individually in 40×40×30 cm compartments, in a lighted and quiet room. Locomotor activity was assessed automatically in a computerized actimeter (Versamax, AccuScan Instruments Inc, Ohio, USA), which monitored the horizontal displacements and vertical movements, including vertical time and vertical activity. The responses were expressed as the total number of beams crossed (horizontal and vertical activity) and as time passed vertically (vertical time) by rats during six consecutive 10-minutes periods.

#### CatWalk gait analysis

CatWalk gait analysis was performed as described elsewhere [37]. Briefly, the animals crossed a horizontal glass runway equipped with a standard CCD camera connected to a computer with the CatWalk software (Noldus Information Technology, Wageningen, Netherlands). Animals were tested for at least three uninterrupted runs per animal. Coordination and different individual paw parameters were assessed to quantify functional recovery.

#### Histological studies

All the histological analyses were performed blindly.

In order to verify the correlation between electrophysiological, locomotor and anatomical recovery, we performed immunostaining for axonal regrowth, astrocyte reactivity, glial scar and inflammatory response. Briefly, anaesthetized rats were transcardially perfused first with ice-cold PBS for 2 min and then 4% paraformaldehyde for 15 min. Spinal cord segments were collected and post-fixed in 4% paraformaldehyde for 24 h at 4°C. After cryopreservation in 30% sucrose at 4°C, spinal cord tissue was cut into longitudinal sections (20 µm) on a cryostat. Following two rinses in PBS, sections on slides were permeabilized using PBS containing 0.2% Triton X-100 for 30 min. Sections were incubated 30 min at room temperature with the corresponding primary antibody: anti-GFAP (1/200, Chemicon, Billerica, MA), anti-neurofilament H (1/200, Chemicon), anti-Neurocan (1/200, Chemicon) and anti-ED1 (1/200, Chemicon). To assess the specificity of the staining, positive and negative controls have been used prior to the experiments. In addition for each experiment, to measure background, slides without primary antibodies were used. Following primary antibody incubation, sections for ED1 and neurocan stainings were incubated 30 min at room temperature with the PE-conjugated anti-mouse antibody (1/500 BD Biosciences). GFAP and neurofilament antibodies were conjugated with Alexa Fluor® 488. Images were analyzed and captured with a fluorescence microscope equipped with apotome (100×; Axioscope; Zeiss). Then, immunodensitometry were performed by outlining areas measuring 1.410^6^ pixels (2.15 mm^2^) for GFAP, neurofilament, neurocan and ED1 stainings using ImageJ software (NIH software). For this, number of positive pixels was counted using the measure tool of ImageJ based on the threshold defined with control animals (without surgery) as described by Stamegna et al. [19].

To address the localization of transplanted OM-OECs into the spinal cord, GFP-Neurofilament co-staining was performed using anti GFP antibody (Santa Cruz Biotechnology, CA). Stainings were performed as described previously.

#### MRI

Experiments were performed 7 days after spinal cord transection in 4 other rats for Md, primary OB and purified OM treated groups (140–160 g) on a 7 Tesla Pharmascan magnet (Bruker, Germany) in anesthetized rats (1.5% isoflurane in O_2_/N_2_O 33%/67%). Sagittal T2-weighted images were acquired with relaxation enhancement (RARE) with an accelerating factor of 4 (TR = ∼ 6000 ms, TE = 40 ms, slice thickness = 0.5 mm, planar resolution of 100 µm * 100 µm, acquisition time ∼ 35 minutes). Image analysis was performed using Image J software.

### Statistics

Prism software (Graphpad Software, La Jolla, CA.) was used for statistics. All data, except for Catwalk analysis, are presented as means ± SEM. Nonparametric test was used (Kruskal-Wallis), followed by a post test (Dunns), to identify differences between the groups. For CatWalk analyses data are presented as percentage of runs succeed and failed and a Fisher’s exact test was performed. Differences were accepted as statistically significant at *p<0.05, **p<0.01, or, and ***p<0.001.

## Results

### Electrophysiological Studies

**Figure 3:**
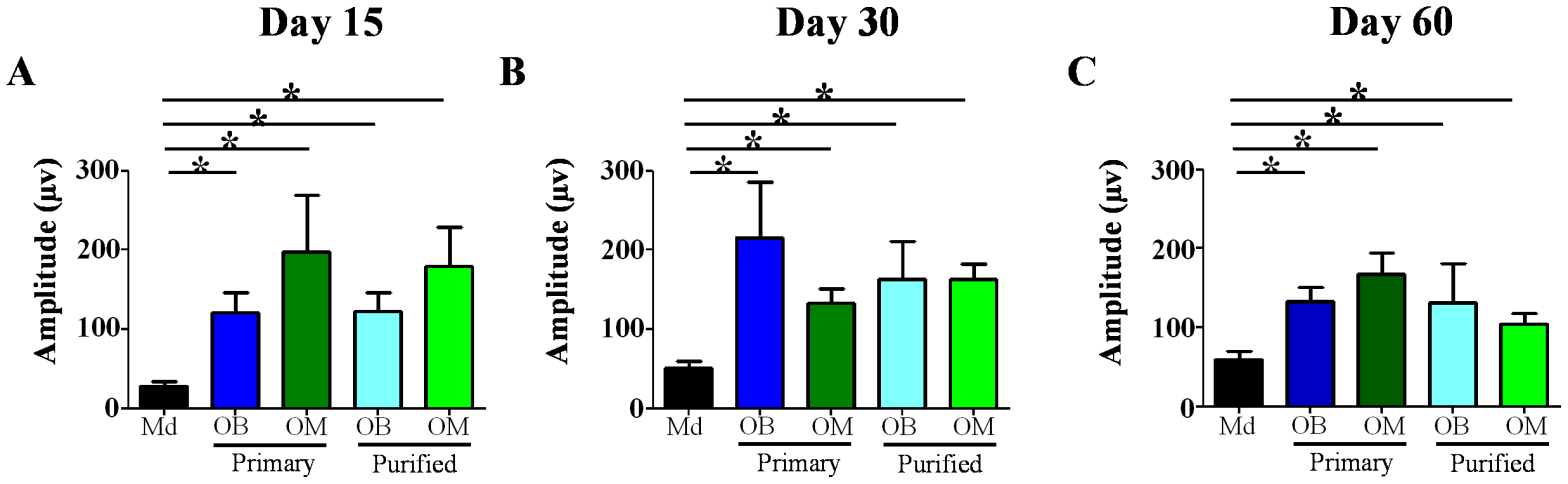
Analysis of electrophysiological activities. Measurement of cord dorsum potentials (**A–C**) shows that transplantation of OECs leads to increase potential amplitude from all OECs treated groups at all-time points studied (**A–C**). Md: Medium, OB: Olfactory Bulb, OM: Olfactory Mucosa. Statistical comparisons were performed using a nonparametric test (Kruskal-Wallis). Mean±SEM represents data from 10 rats per group. *: p value<0.05.

Stimulations were performed above the lesion site and CDP were measured using electrodes placed in gastrocnemius muscles. These analyses showed that at D15, all OECs treated groups presented a significant improvement of the CDP amplitudes in comparison to the Md group (p<0.05) (Figure 3A). Increase of CDP amplitudes persisted for the full time duration of the experiment for all OECs treated groups. In fact, all OECs treated groups showed at D30 and D60 significantly higher CDP amplitudes in comparison to the Md group (p<0.05) (Figures 3B and C). For this analysis no difference could be observed between the different OECs treated groups (Figure 3). These results demonstrate that transplantations of different types of OECs equally induce electrophysiological recovery from early (D15) to late time points (D60).

### Behavioral Studies

#### Measurement of locomotor activity

**Figure 4:**
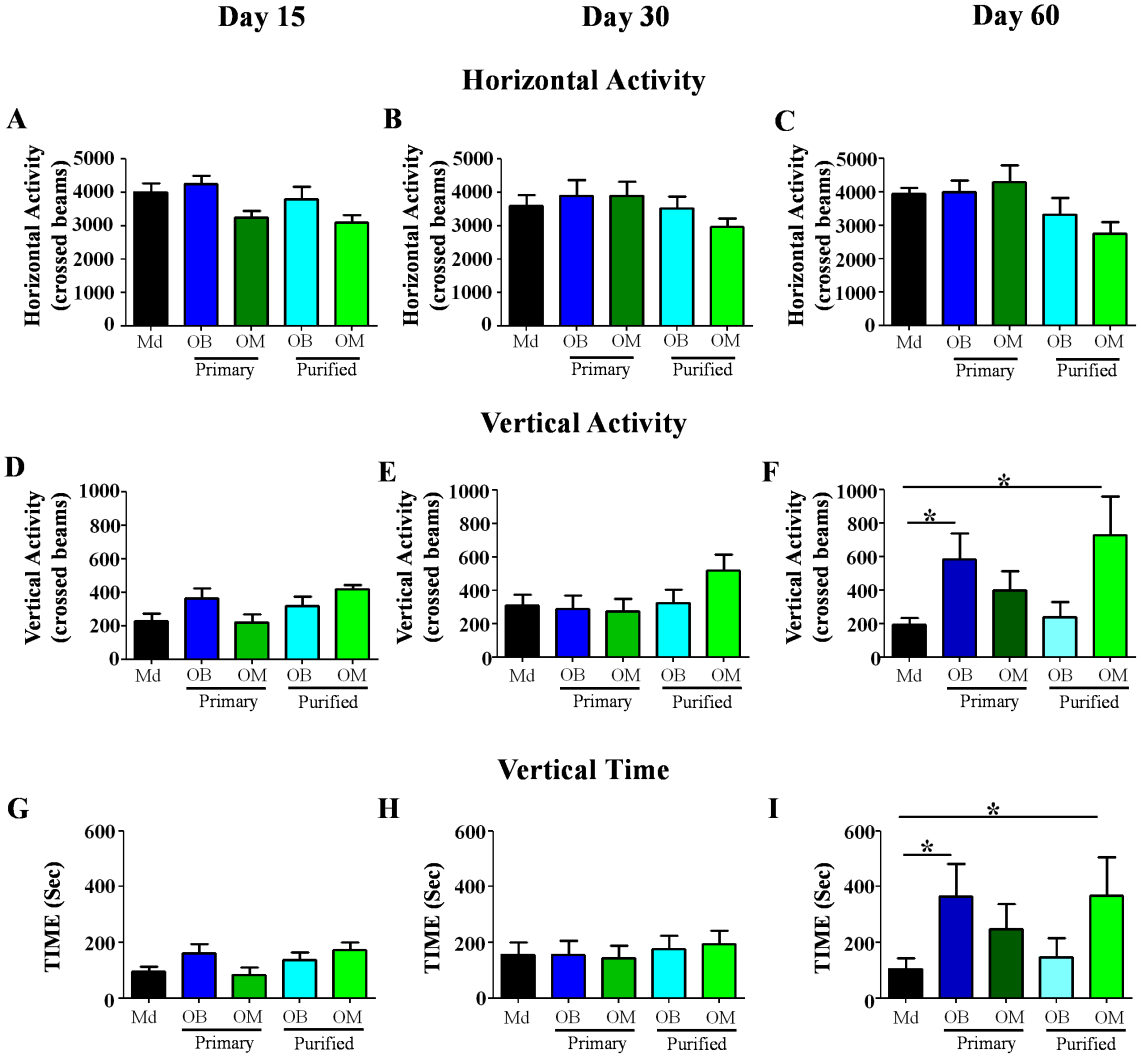
Analysis of locomotor activities. **A–C**: horizontal activity **D–F**: vertical activity and **G–I:** vertical time. Measurement of locomotor activity (**A–C**) reveals that there is no significant difference between the groups for horizontal activity at all-time points. This analyses shows that transplantation of Primary OB and Purified OM improve vertical activity (**F**) and vertical time (**I**) at 60 days. Md: Medium, OB: Olfactory Bulb, OM: Olfactory Mucosa. Statistical comparisons were performed using a nonparametric test (Kruskal-Wallis). Mean±SEM represents data from 10 rats per group. *: p value<0.05.

Measurement of horizontal activity revealed that during the full duration of the experiment all groups presented no significant difference for this parameter (Figures 4A–C). These results demonstrate that all groups have the same ability to move and to explore. At D15 and D30 all the groups showed no significant difference for vertical activity and vertical time (Figures 4D–E and G–H). However, at D60, Primary OB and Purified OM treated groups presented a significant higher vertical activity (p<0.05) in comparison to the Md treated group (Figure 4F). Similarly, at D60 Primary OB and Purified OM treated groups presented a significantly higher vertical time (p<0.05) in comparison to the Md treated group (Figure 4I). These results indicate that Primary OB and Purified OM can promote locomotor activity, in particular ability to stand up and to maintain a standing position, at later time (D60).

#### CatWalk gait analysis

This analysis revealed that for all time points analyzed there was no significant differences between all the groups, due to a high intra-group dispersion (data not shown). However, contingency analysis was performed. For this, we have counted all the runs performed by the animals during the three distinct periods (D15 to D60). Then, we have counted the number of successes and failures based on the ability of animals to have walking into the corridor and we have determined the ratio of successes and failures for each group. This analysis showed that Primary OB, Purified OB and Purified OM treated groups displayed a significantly higher rate of runs succeed in comparison to the Md treated animals. Indeed, 0% of the Md treated group has been walking during all the runs, in comparison, 21.6% of the Primary OB (p<0.05), 26% of the Purified OB (0.01<p<0.001) and 36.7% of the Purified OM treated animals (p<0.001) were able to walk into the corridor during all the runs (Table 1). Moreover, Purified OM treated group showed a significantly higher probability to walk in comparison to the Primary OM treated animals (p<0.05).These results demonstrate that Primary OB, Purified OB and Purified OM treated groups, but not Primary OM treated group can improve ability to walk.

**Table 1:** Catwalk analysis.

	Md	Primary OB	Primary OM	Purified OB	Purified OM
Run succeed (%)	0–0/25	21.6*–8/37	10.3–3/29	26**–8/31	36.7*** #–11/30
Run failed (%)	100–25/25	78.4–29/37	89.7–26/29	74–23/31	63.3–19/30

Catwalk analysis reveals that Primary OB, Purified OB and Purified OM animals show a significant higher probability to walk (based of the higher rate of runs succeed). Percentage (%) is based on run succeed or failed during all the time points. Total number of runs succeed and failed are presented for each group. Md: Medium, OB: Olfactory Bulb, OM: Olfactory Mucosa. Statistical comparisons were performed using a Fisher’s exact test.

* : p value<0.05;

** : p value<0.01,

*** : p value<0.001 and #: p value<0.05.

* represents significant differences between transplanted groups (Primary OB, Purified OB and Purified OM) and Md group.

# represents significant difference between Purified OM and Md groups.

### Histological Analysis

**Figure 5:**
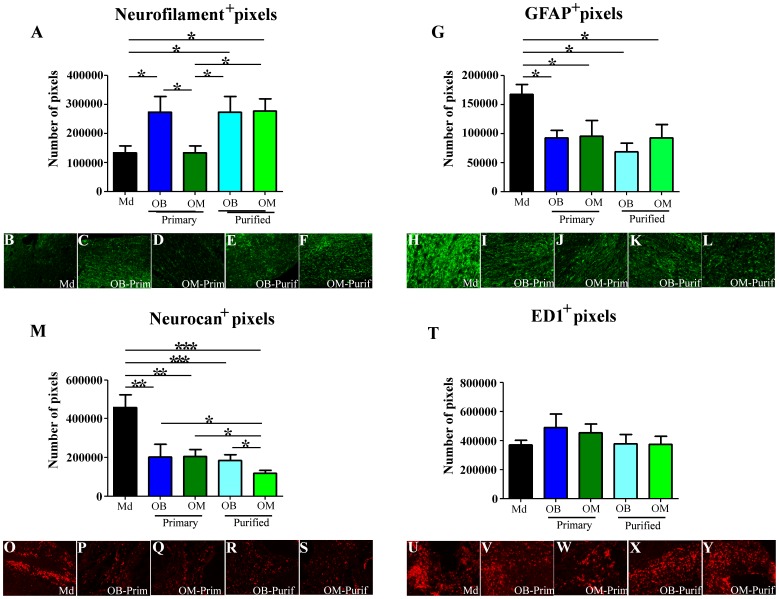
Histological analyses. Histological analyses on longitudinal sections were performed for neurofilament (**A**), GFAP (**G**), Neurocan (**M**) and ED1 (**T**). These analyses reveal that Primary OB, Purified OB and Purified OM OECs increase axonal regrowth (neurofilament) (**A**), reduce astrocyte reactivity (GFAP) (**G**) and glial scar formation (Neurocan) (**M**). At the same time this analysis reveals that OECs transplantations had no influence on monocytes/microglia infiltration (ED1) (**T**). Typical illustrations, are shown for each group for neurofilament (**B–F**), GFAP (**H–L**), neurocan (**O–L**) and ED1 (**U–Y**). Md: Medium, OB: Olfactory Bulb, OM: Olfactory Mucosa. Statistical comparisons were performed using a nonparametric test (Kruskal-Wallis). Mean±SEM represents data from 10 rats per group. *: p value<0.05; **: p value<0.01 and ***: p value<0.001.

All the histological analyses were performed on longitudinal sections. At D60, histological analyses on an equivalent area revealed that Primary OB, Purified OB and Purified OM treated groups presented a significantly higher staining for neurofilament, used as a marker of axonal regrowth, in comparison to Md treated group (p<0.05) (Figure 5A). Primary OM treated group did not show difference with Md treated group (Figure 5A). Representative pictures are presented for each group for neurofilament staining (Figures 5B–F). Conversely, similar histological analyses revealed that all OECs treated groups presented a significantly lower staining for GFAP, used as a marker of astrocyte reactivity, in comparison to Md treated group (p<0.05) (Figure 5G) [38]. Representative pictures are presented for each group for GFAP staining (Figures 5H–L). We also performed neurocan staining, an extracellular matrix (ECM) protein present in the glial scar, and showed that all OECs treated groups presented a significant reduction for this staining (0.01<p<0.001for Primary OM and OB treated groups and p<0.001 for Purified OB and OM treated groups) in comparison to Md treated group (Figure 5M). Of interest, Purified OM treated group showed the greatest reduction of neurocan staining (p<0.05). Representative pictures are presented for each group for neurocan staining (Figures 5O–S).

At D60, there was no difference in ED1 staining, used as a marker of activated microglia, monocytes and macrophages, between all groups. In fact, all groups presented a high ED1+ invasion into the spinal cord parenchyma (Figure 5T). Representative pictures are presented for each group for ED1 staining (Figures 5U–Y).Complementary analyses were performed in order to determine the potential presence of distinct subpopulations of macrophages (M1 and M2) into the ED1+ cells. For this, histological analyses based on MMP9-ED1 co-staining were done, but no difference could be observed between the groups (data not shown). We further confirmed these results by an *in vitro* approach using macrophages (NR8383 cells) cultured with conditioned medium obtained from OM and OB-OECs [7]. Quantification of IL-12 and IL-10 secretion showed that conditioned medium from OM or OB-OECs had no effect on macrophage phenotype in culture (data not shown).

Altogether, these results demonstrate that all OECs treated groups presented a reduction of GFAP (astrocyte reactivity) and neurocan (glial scar component) stainings in comparison to Md treated group and in particular Purified OM treated group displayed a significantly greater reduction of neurocan expression in comparison to all the other OECs treated groups. However it could be demonstrated that Primary OB, Purified OB and Purified OM treated groups and not Primary OM treated group showed an increase of the axonal regrowth (neurofilament) in comparison to Md treated group.

### MRI Analysis

**Figure 6:**
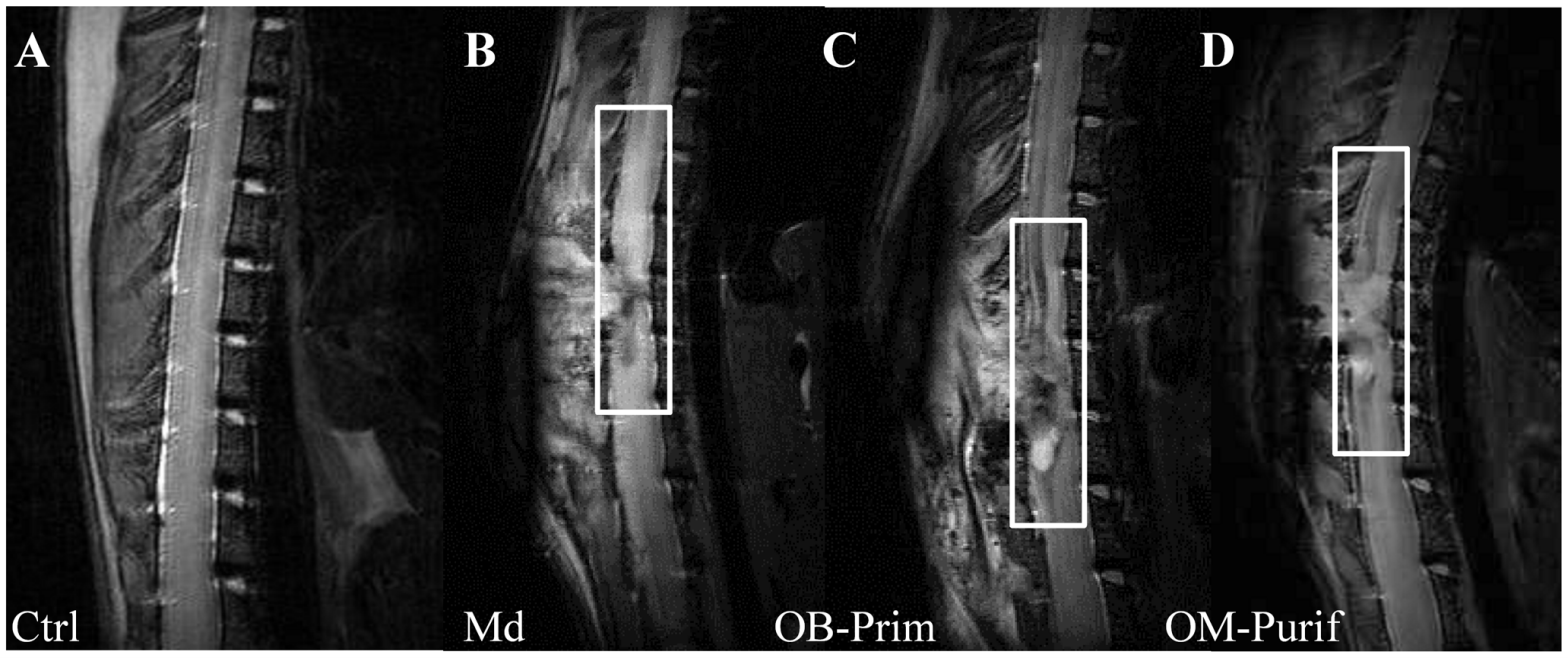
MRI analyses. MRI analyses revealed that Primary OB (**C**) and Purified OM OECs (**D**) decreased inflammatory infiltrate and edema 7 days after transplantation. Boxes represent areas of injury. Typical illustrations are shown for control (**A**), Md (**B**), Primary OB (**C**) and Purified OM OECs (**D**). Ctrl: Control, Md: Medium, OB: Olfactory Bulb, OM: Olfactory Mucosa. Illustrations are representative of four different animals for each group.

To further address the inflammatory infiltrate and formation of the glial scar at early time point after surgery and/or cellular transplantation, we performed MRI experiments after 7 days. For this analysis 4 other rats of Md, Primary OB and Purified OM treated groups, which showed higher potential to promote recovery, were used. Transplanted animals (Figures 6C and D) presented a reduction of the signal intensity (delimited by white areas) in comparison to the hyperintense sequences of the Md treated animals (Figure 6B). This result based on T2* IRM sequences indicates that OEC transplantations reduce inflammatory infiltrate and edema into the spinal cord parenchyma at early time. Animals without surgery served as control (Figure 6A).

### Tracking of GFP+ OM-OECs

**Figure 7:**
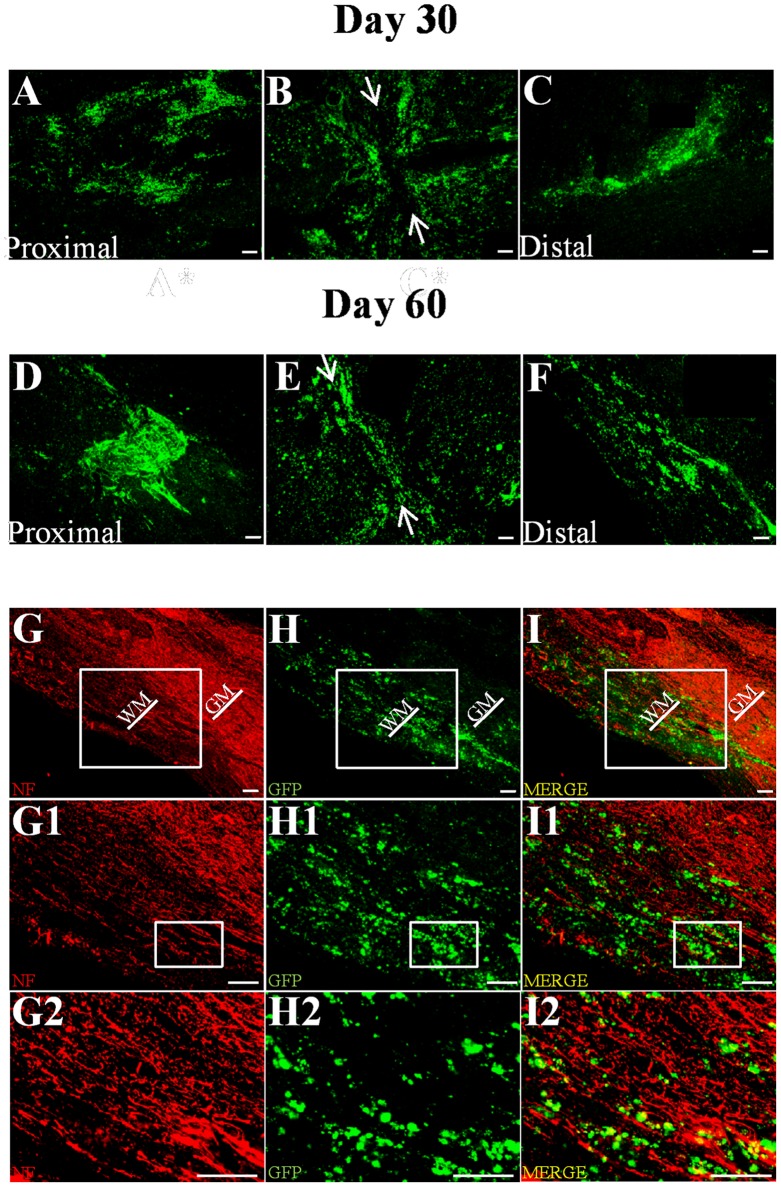
Tracking of GFP+ OM-OECs. Tracking of GFP+ OM-OECs performed on longitudinal sections revealed that 30 (**A–C**) and 60 (**D–F**) days after transplantation, OECs could be found at the injury site (**B and E**) and both proximally (**A and D**) and distally (**C and F**) to the injection site. White arrows represent the lesion site. GFP/neurofilament co-staining shows that OECs are closely associated to neurofilament+ fibers (**G–I2**), 30 and 60 days after surgery. Scale bar = 100 µm. WH = White Matter and GH = Grey matter. Illustrations are representative of three different animals for each time point.

For this analysis, 6 other rats for Purified OM treated group, which constituted the most relevant group for clinical use, were used. Tracking of GFP+ OM-OECs was performed 30 and 60 days after surgery on 3 rats for each time point. Histological analyses, based on longitudinal sections, revealed that a similarly high number of GFP+ cells could be found at the lesion site and both distally (Figures 7C and F) and proximally (Figures 7A and D) to the injection site at both time points in all the rats transplanted.

Image analysis demonstrated that OECs were spread on a distance of 1.5 cm and on a maximal distance of the injection sites of around 0.35 cm. It should be noted that the lesion site did not show gap 60 days after transplantation (white arrows) (Figure 7E) in contrary to 30 days after transplantation (Figure 7B).

To address the localization of OECs into the spinal cord, GFP-Neurofilament co-staining was performed and shows that OECs were closely associated to neurofilament 30 and 60 days after surgery (Figures 7G–I2).

Altogether, these results demonstrate that OM-OECs can survive up to 60 days, migrate and are associated with regrowing axons in the lesioned spinal cord.

## Discussion

Numerous reports have attempted to address the role of OECs transplantations after SCI using different experimental paradigms [13], [38]–[42]. However, none of these studies has directly compared the therapeutic benefit of OM and OB-OECs, from primary or purified cultures, by electrophysiological, functional and histological analyses. To our knowledge, this is the first report which described by a multi-parametric approach the effects of OECs from different sources in a severe SCI model.

For our study we have chosen a complete transection model due to the fact that this model is easily reproducible and induces a permanent disability on lesioned animals [43], [44]. To ensure of the completeness and the reproducibility of the section all the surgeries have been performed by the same operator (A.M) and preliminary animals have been used in which we have performed immediate electrophysiology analyses after transection for demonstrating the abolition of the electrical signal.

Locomotor activity and catwalk tests have been chosen preferentially to BBB to monitor functional recovery, because they are computer-assisted analysis and allow to collect quantitative data with a great precision. Indeed, lomomotor activity analyses revealed that all the lesioned animals have the same horizontal activity (ability to move), however OECs transplantations specifically induce stand up ability in Primary OB and Purified OM treated animals. These results prove that Md animals were specifically not able to stand up (Figure 4).

Our results demonstrate that transplantation of OECs constitutes a very promising therapy for SCI independently of their source. Indeed, OECs induced electrophysiological and functional recovery (Figures 3 and 4 and Table 1), reduced astrocyte reactivity and glial scar (based on neurocan expression) and improved axonal regrowth (Figure 5). Interestingly, ED1 staining revealed that OECs seem to have no effect on macrophage/microglia response 60 days after SCI (Figure 5). To further address ability of OECs to modulate processes and formation of the glial scar at early time point after surgery and/or cellular transplantation MRI analyses were performed. Seven days after surgery the MRI sequences in treated groups showed a reduction of the signal intensity in comparison to the untreated group. These results could be interpreted as a reduction of the inflammatory infiltrate and edema or as a reduction of the cystic cavities. Both of these hypotheses seem to indicate a decrease of the inflammatory processes 7 days after surgery in transplanted groups (Primary OB and Purified OM groups). Our results show equally that electric recovery (based on measurement of cord dorsum potentials) appeared early after transplantation (D15) in treated groups. However, this recovery is followed by later functional recovery in primary OB and purified OM-OECs treated groups (D60). These results could be probably explained by the fact that to induce functional recovery, neurons have not only to regrowth but also to form connections to constitute neuronal network.

Although no significant difference about functional recovery can be showed between OB-Primary and OM-Purified groups, our study highlights that OECs obtained and purified from OM present the best benefit/risk ratio. This result is a fundamental point for a future human clinical application, due to the fact that OM is the more accessible source for OECs.

In our study, as previously reported, purification of primary cultures has no effect in case of transplantation of OECs obtained from OB (Figures 3–5 and Table 1) which can be explained in several ways [24]. First, deprivation of microenvironment induces a loss of the two distinct subpopulations of OECs present in primary OB cultures, leading to a decrease in the recovery of the treated animals [21]. Secondly the rate of OECs is around 70% in primary OB cultures which might not be a limiting factor for functional and anatomical recovery in our SCI model. Finally, the “fibroblast-like” cells present in primary OB cultures may play an important role for the integration of OECs in the spinal cord [28], [45]. Indeed, a recent study has underlined the main role of fibroblasts on SC for axonal regrowth after Wallerian degeneration in peripheral nerve injury (PNI) [46]. It could be reasonably envisaged, due to the common origin of SC and OECs, that “fibroblast-like” cells have the same influence on OECs, even in the case of transplantation in a lesioned spinal cord [14], [47].

In contrast, purification of OECs from OM cultures improves significantly both functional and anatomical recoveries which is highly relevant for future clinical applications. Several hypotheses could explain the differences observed between primary and purified cultures upon transplantation. First, primary OM cultures have a relatively low content of OECs (15%) which could explain the poor functional recovery of this group. Nevertheless, several studies have reported benefic functional effects after transplantation of low dose of OECs [16], [48]. Another hypothesis is that contaminating cells present in primary OM cultures inhibit axonal regrowth. In our study, histological analyses revealed that Primary OM treated group displayed no difference according to neurofilament staining in comparison to Md group, whereas in the same time Purified OM treated group showed a significant increase for this parameter (p<0.05) (Figure 5). We previously have reported that transplantation of primary OM cultures after PNI induces functional recovery without improvement of axonal regrowth [23]. The microenvironment present in primary OM cultures could also induce a higher mortality after cellular transplantation. One last hypothesis is that the purification method used, based on privative medium, might induce modification or selection of specific cell subpopulations. A recent study has reported that primary OM cultures contain distinct populations of cells, assumed to be OECs, therefore serum deprivation may select specifically one of them [49]. Complementary studies should be performed to address this specific point.

Previous studies have compared OM and OB-OECs in both *in vitro* cultures and after PNI *in vivo* [23], [50], [51]. They have shown some differences such as distinct ability to induce axonal regrowth, to modulate ECM and to interact with primary olfactory neurons [23], [50], [51]. In our present study, no major differences in functional recovery after transplantation of OM and OB-OECs were observed upon SCI (Figures 3–5). However, our histological analyses revealed a significant difference between these groups. In fact, neurocan staining showed that Purified OM group presented a significant reduction of this parameter in comparison to Primary OB and Purified OB treated groups (p<0.05) (Figure 5). Recently, it was discovered that ADAMTS-4 could play a major role in degradation of the glial scar. Indeed, chondroitin sulfate proteoglycans (CSPG) are a major component of the glial scar with axonal growth inhibitory properties. Recently, it was shown that ADAMTS-4 could degrade CSPG as neurocan, brevican and phosphacan *in vitro* and *in vivo* and that local administration of ADAMTS-4 can promote functional recovery after SCI [4]. We have previously shown that *ADAMTS-4* is more highly expressed by OM-OECs than by OB-OECs *in vitro* [20]. We have further confirmed this result at the protein level (data not shown). Previous studies have demonstrated the ability of OM-OECs to modulate the ECM [35], [52]. Therefore, complementary studies on the capacity of OM-OECs, in particular the potential role of ADAMTS-4 to regulate glial scar, should be conducted.

Visualization of GFP labeled OM-OECs showed that they were present and closely associated to neurofilament+ fibers (Figure 6). We showed that OM-OECs could migrate into the injured spinal cord and survive for up to 60 days. OM-OECs, in our model, migrated up to 3.5 mm, a distance close to previously reported genetically modified SC (4.4 mm) [8]. These results highlight the potential of OM-OECs to migrate and to integrate into the injured spinal cord.

Altogether, the accessibility and the recovery properties of OECs allow to propose them as a future relevant biotherapy for spinal cord repair. Several types of cellular transplantations have been tested to promote SCI recovery in rodent models, however few of these could be proposed for clinical use. In fact for a human application, the cells must be easily sampled and cultured even from adult, must present weak risk of teratogenicity and must induce functional recovery. OECs have all these advantages and criteria. Specifically, OM-OECs can be obtained from nasal biopsies and cultured with high purity and present a high benefit/risk balance due to the fact that OECs are differentiated cells, are stable even after long term cultures from adult primates and induce functional and anatomical recoveries [29], [34].

We have demonstrated the potential of different OECs sources. However a better molecular understanding on the culture effect and subpopulations will further strengthen the clinical potential and use of OECs for SCI.
